# Two patients with COVID-19 and a fever-induced Brugada-like electrocardiographic pattern

**DOI:** 10.1007/s12471-020-01459-1

**Published:** 2020-07-08

**Authors:** S. W. E. van de Poll, C. van der Werf

**Affiliations:** 1 Department of Cardiology, Franciscus Gasthuis and Vlietland Hospital, Rotterdam, The Netherlands; 2 Heart Center, Department of Clinical and Experimental Cardiology, Amsterdam Cardiovascular Sciences Amsterdam, Amsterdam UMC, University of Amsterdam, Amsterdam, The Netherlands

**Keywords:** COVID-19, Fever, Brugada syndrome

## Abstract

Febrile states may unmask certain Brugada syndrome patients and precipitate ventricular arrhythmias. Here we describe two patients with COVID-19 who developed a fever-induced type 1 Brugada electrocardiographic pattern. Both patients did not show any ventricular arrhythmias during admission. These and previously published cases suggest that the threshold to run an ECG should be low in febrile patients with suspected COVID-19, because these patients are potentially at risk for developing proarrhythmic complications.

## What’s new?

- A febrile state due to COVID-19 may unmask a type 1 Brugada-like electrocardiographic pattern.
- These patients are potentially at risk for developing proarrhythmic complications.
- The threshold to run an ECG should be low in febrile patients with suspected COVID-19.

## Patient 1

A 58-year-old man with a history of atrioventricular nodal re-entry tachycardia treated with catheter ablation, Bell’s palsy and chronic facial pain presented to the emergency department with a 6-day history of fever, cough and shortness of breath. Despite being treated with amoxicillin for four days the shortness of breath had increased. The patient also complained of chest pain radiating to the back and abdominal pain on the right side. He did not report a history of syncope, and there was no relevant family history.

**Fig. 1:**
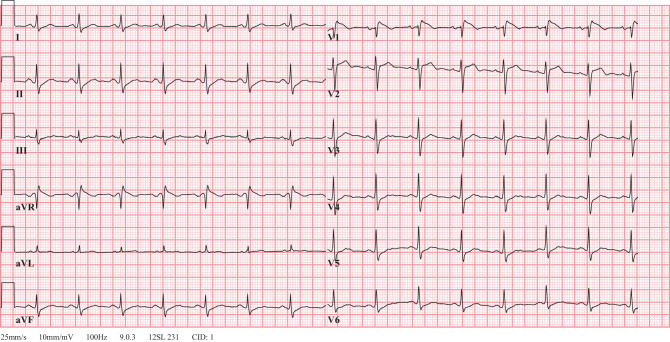
Patient 1’s initial 12-lead electrocardiogram in the emergency department

On examination, the patient had a fever (39.0 °C) and tachypnoea (respiratory rate 30 breaths per minute) with otherwise stable vital signs. Chest radiography showed a possible consolidation in the left lower lobe. Laboratory data demonstrated an elevated C‑reactive protein level (64 mg/l). The electrocardiogram (ECG) showed sinus rhythm 88 beats per minute and a Brugada type 1‑like ECG pattern in lead V1 (Fig. 1).

**Fig. 2:**
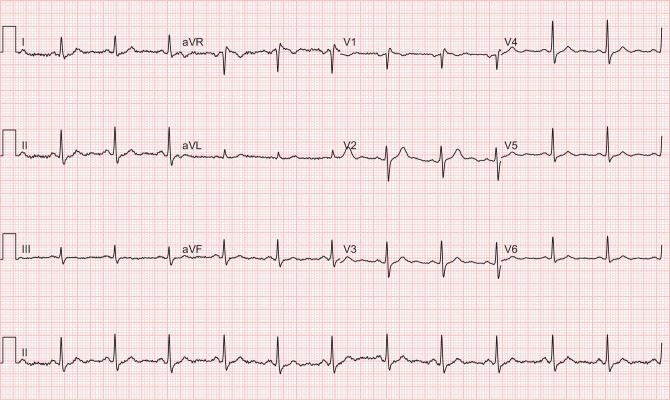
Patient 1’s repeat 12-lead electrocardiogram with resolution of fever

The patient was placed on airborne isolation precautions in a dedicated coronavirus disease-2019 (COVID-19) unit with telemetry monitoring and received antipyretic therapy (paracetamol 1000 mg QID). He tested positive for COVID-19. He required minimal supplemental oxygen to maintain arterial saturation. There were no arrhythmias. After six days of admission, the ECG showed sinus rhythm 70 beats per minute with resolution of the Brugada-like ECG pattern (Fig. 2) and the patient was discharged.

## Patient 2

A 40-year-old man without relevant past medical history presented to the emergency department with a 5-day history of fever (39.4 °C), chills and cough. In addition, he had experienced a syncopal episode 3 days prior to his presentation. The syncope occurred in the kitchen shortly after the patient got out of bed, without any prodromal symptoms and with a very brief loss of consciousness. Twenty years ago, he experienced a similar event while being febrile. There was no relevant family history.

**Fig. 3:**
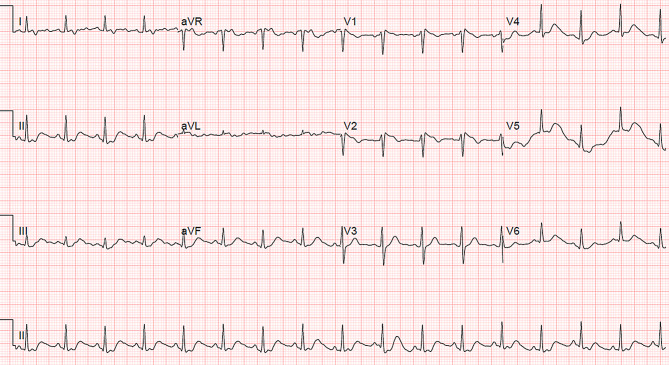
Patient 2’s initial 12-lead electrocardiogram in the emergency department

On examination, the patient was febrile (38.6 °C) with otherwise stable vital signs. Chest radiography showed a left-sided and possible right-sided consolidation. Laboratory data demonstrated an elevated C‑reactive protein level (100 mg/l) and lactate dehydrogenase (508 U/l) and lymphopenia (0.8 × 10^9^/l). The electrocardiogram showed a sinus tachycardia of 100 beats per minute and a borderline Brugada type 1‑like ECG pattern in lead V1 (Fig. 3).

**Fig. 4:**
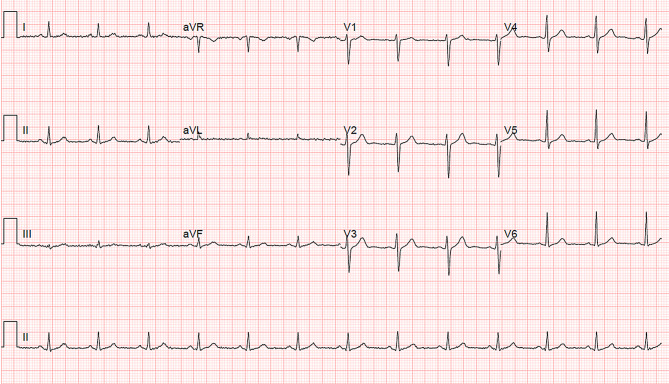
Patient 2’s repeat 12-lead electrocardiogram with resolution of fever

The patient was placed on airborne isolation precautions in a dedicated COVID-19 unit with telemetry monitoring and received antipyretic therapy (paracetamol 1000 mg QID). He tested positive for COVID-19. The patient’s fever improved and the ECG showed a sinus rhythm of 78 beats per minute and resolution of the Brugada-like ECG pattern (Fig. 4). The patient continues to be admitted and no arrhythmias have been observed.

## Discussion

Febrile states may unmask certain Brugada syndrome patients and precipitate ventricular arrhythmias [1, 2]. In a study by Adler and colleagues including 402 patients with fever, the prevalence of a Brugada type 1 ECG pattern was 2% [3]. Because fever is one of the main symptoms of COVID-19, it is conceivable that a significant number of new patients with asymptomatic Brugada syndrome will be identified during the COVID-19 pandemic. However, to our knowledge only two other patients with a COVID-19-induced Brugada-like ECG pattern have been reported [4, 5]. The first patient presented with chest pain [4],the second patient presented with a syncopal event [5]. Both patients did not show any ventricular arrhythmias during admission.

In both patients in this report, heart rate at admission was significantly higher than during follow-up when resolution of the Brugada type 1 ECG pattern occurred. Exercise is known to aggravate the ECG phenotype in patients with Brugada syndrome [6]. Therefore, it is possible that, besides a direct effect of fever on the underlying pathophysiological substrate (e.g. loss-of-function of the voltage-gated sodium [Na_v_]1.5 channels), the fever-induced heart rate increase contributed to the unmasking of the Brugada type 1 ECG pattern.

A study in 112 patients with a fever-induced Brugada type 1 ECG pattern showed that 26% carried a *SCN5A* mutation and 80% had a positive sodium channel blocker challenge [2]. They found that the risk of ventricular fibrillation during follow-up was 0.9%, which is comparable with the risk in an asymptomatic individual with a spontaneous Brugada type 1 ECG [7].

Both patients described in this case report will have follow-up, including genetic testing. However, in our opinion an implantable cardioverter defibrillator is not indicated in these patients. Patient 1 had an obvious fever-induced Brugada type 1‑like ECG pattern, but was asymptomatic. In patient 2 it is possible that the syncope was non-arrhythmic and it is debatable whether the fever-induced Brugada pattern fulfils the diagnostic criteria of a type 1 pattern. In addition, fever can be effectively treated with antipyretic therapy, which probably reduces the risk of ventricular arrhythmias. Recently, Wu and colleagues described the potential COVID-19-associated risks in known patients with Brugada syndrome [8]. They recommended that febrile higher-risk patients, defined as patients without an implantable cardioverter defibrillator who 1) have a pathogenic or likely pathogenic *SCN5A *mutation, 2) are aged <26 or >70 years, or 3) have a spontaneous Brugada type 1 pattern or cardiac syncope, attend an emergency department. These recommendations now also apply to patient 2.

In conclusion, the two previously published case reports [4, 5] and the two cases described in this report suggest that the threshold to run an ECG should be low in febrile patients with suspected COVID-19, because these patients are potentially at risk for developing proarrhythmic complications.
